# Supplementary material for: Epilepsy and Language Development in 8–36-Month-Old Toddlers with Tuberous Sclerosis Complex
Source: J Clin Med. 2022 Aug 4;11(15):4564. doi: 10.3390/jcm11154564 (PMC9369686; doi:10.3390/jcm11154564)
Supplement: Supplementary file 1 [file jcm-11-04564-s001.zip › jcm-1794771-supplementary.pdf]

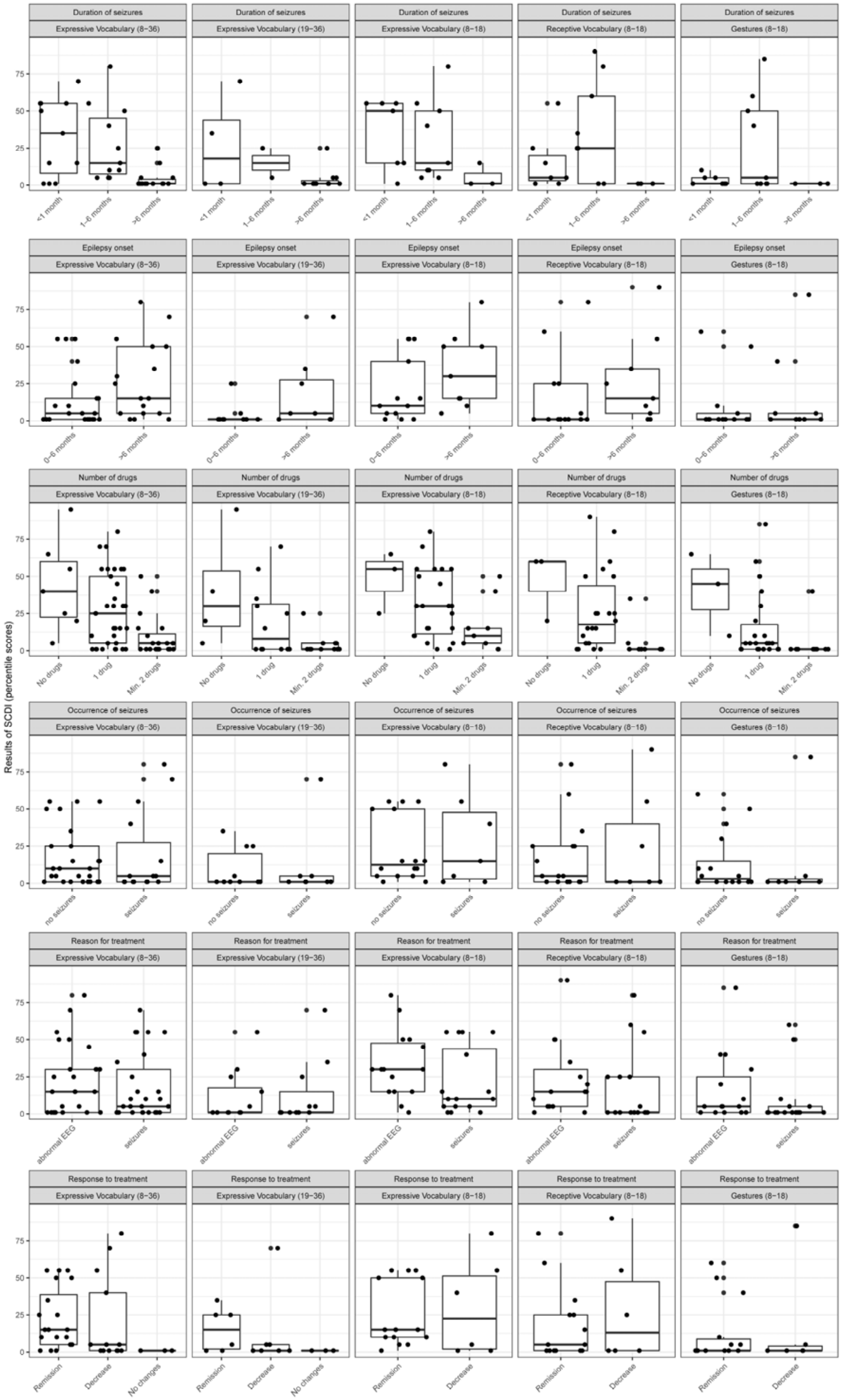

Results of SCDI (percentile scores)
Duration of seizures
Expressive Vocabulary (8–36)
Expressive Vocabulary (19–36)
Expressive Vocabulary (8–18)
Receptive Vocabulary (8–18)
Gestures (8–18)
<1 month
1–6 months
>6 months
Epilepsy onset
0–6 months
>6 months
Number of drugs
No drugs
1 drug
Min. 2 drugs
Occurrence of seizures
no seizures
seizures
Reason for treatment
abnormal EEG
seizures
Response to treatment
Remission
Decrease
No changes
0
25
50
75

Figure S1: Quartile distribution of results: Duration of seizures, response to treatment and number of drugs. The Y axis indicates the SCDI; percentile score. On the X axis there are names of groups of individual variables.
